# Genome-wide association analysis reveal the genetic reasons affect melanin spot accumulation in beak skin of ducks

**DOI:** 10.1186/s12864-022-08444-5

**Published:** 2022-03-26

**Authors:** Hehe Liu, Jianmei Wang, Jian Hu, Lei Wang, Zhanbao Guo, Wenlei Fan, Yaxi Xu, Dapeng Liu, Yunsheng Zhang, Ming Xie, Jing Tang, Wei Huang, Qi Zhang, Zhengkui Zhou, Shuisheng Hou

**Affiliations:** 1 State Key Laboratory of Animal Nutrition; Key Laboratory of Animal (Poultry) Genetics Breeding and Reproduction, Ministry of Agriculture and Rural Affairs, Institute of Animal Science, Chinese Academy of Agricultural Sciences, Beijing, 100193 China; 2 Farm Animal Genetic Resources Exploration and Innovation Key Laboratory of Sichuan Province, College of Animal Science and Technology, Sichuan Agricultural University, Chengdu, 613000 China

**Keywords:** Beak skin melanin spot, Duck, GWAS, Genetic

## Abstract

**Background:**

Skin pigmentation is a broadly appearing phenomenon of most animals and humans in nature. Here we used a bird model to investigate why melanin spot deposits on the skin.

**Results:**

Our result showed that growth age and the sunlight might induce melanin deposition in bird beak skin which was determined by genetic factors. GWAS helped us to identify two major loci affecting melanin deposition, located on chromosomes 13 and 25, respectively. The fine mapping works narrowed the candidate regions to 0.98 Mb and 1.0 Mb on chromosomes 13 and 25. The MITF and POU2F3 may be the causative genes and synergistically affect melanin deposition during duck beak skin. Furthermore, our data strongly demonstrated that the pathway of melanin metabolism contributes to melanin deposition on the skin.

**Conclusions:**

We demonstrated that age and sunlight induce melanin deposition in bird beak skin, while heredity is fundamental. The MITF and POU2F3 likely played a synergistic effect on the regulation of melanin synthesis, and their mutations contribute to phenotypic differences in beak melanin deposition among individuals. It is pointed out that melanin deposition in the skin is related to the pathway of melanin metabolism, which provided insights into the molecular regulatory mechanisms and the genetic improvement of the melanin deposition in duck beak.

**Supplementary Information:**

The online version contains supplementary material available at 10.1186/s12864-022-08444-5.

## Introduction

Skin pigmentation is a broadly appearing phenomenon in most animals and humans. It is related to the health conditions and the age status. In humans, changes in skin color are often associated with age [1]. Although numerous efforts have been made to retard the skin aging process, the skin structure and function declined over time and long-term exposure to ultraviolet (UV) irradiation from the sunlight [2]. Eventually, the skin pigmentation of sun-exposed areas, i.e., face, neck, forearms, or dorsal surface of hands, becomes asymmetrical with age due to continuous sun exposure, and this mottled pigmentation develops a sign of aged skin [3, 4]. However, in sunshade areas of skin, the pigmentation and reactive tanning decrease with age [5, 6]. In birds, skin pigmentation increases with age also, e.g., the female Pekin duck accumulates a large amount of melanin in the beak skin during the breeding period. In poultry production, excessive melanin deposition in the skin directly affects the sensory quality and the desire of consumers to purchase.

The colors of feathers, furs, and skin are mainly determined by melanocytes, which take part in the synthesis of melanin pigments that play an essential role in cosmetic variation, heat regulation, and camouflage and simultaneously give protection against UV radiations from sunlight exposure [7]. Moreover, the accumulation of melanin pigments causes skin pigmentation [8]. In the young adult epidermis, melanocytes are pretty evenly distributed on the body at the rate of 1500/mm^2^ density [9, 10]. However, after 30 years of age, the aging process is tied to a 10 to 20 percent per decade decrease in melanin production by melanocytes [11]. As a result, in aging skin, the asymmetrical distribution of melanocytes caused the formation of melanin spots under constantly sun-exposed.

Skin pigment deposition is also highly inherited and regulated by genetic factors [12]. The α-MSH is a significant inherited factor and acts mainly as an agonist of MC1R. The high incidence of MC1R mutations in individuals with red hair and light skin may be responsible for the reduced response to α-MSH, resulting in decreased melanin production and pigmentation caused by UV exposure [13]. Furthermore, it was shown that the polymorphism of MC1R is associated with ethnic differences in structural pigmentation and different responses to UV irradiation [14, 15].

Birds live most of the time outdoors and are more easily affected by ultraviolet radiation from the sunlight. Therefore, melanin stands out in hair follicles, skin, eyes, feathers, and scales [16]. Pekin duck is a famous white-feathered duck breed globally, and the white feather is caused by genetic albinism [17], which means very little melanin is distributed in feathers and skin. The melanin spots on beak skin have been observed during the breeding period of female Pekin ducks. However, the underlying reason is still unclear. Here, we used duck as a model to investigate why melanin spots were deposited on the beak skin. The potential influencing factors, including age, sunlight exposure, and reproduction abilities, were estimated in ducks. Furthermore, based on a collection of 223 F_2_ ducks crossed by Pekin ducks and mallards, we also performed a genome-wide association study (GWAS) to gain insights into the genetic factors influencing melanin deposition in the beak skin. These works provide insights into the molecular regulatory mechanisms and the genetic improvement of the melanin deposition in duck beak.

## Results and analysis

### Descriptive statistics of phenotypic traits

**Fig. 1:**
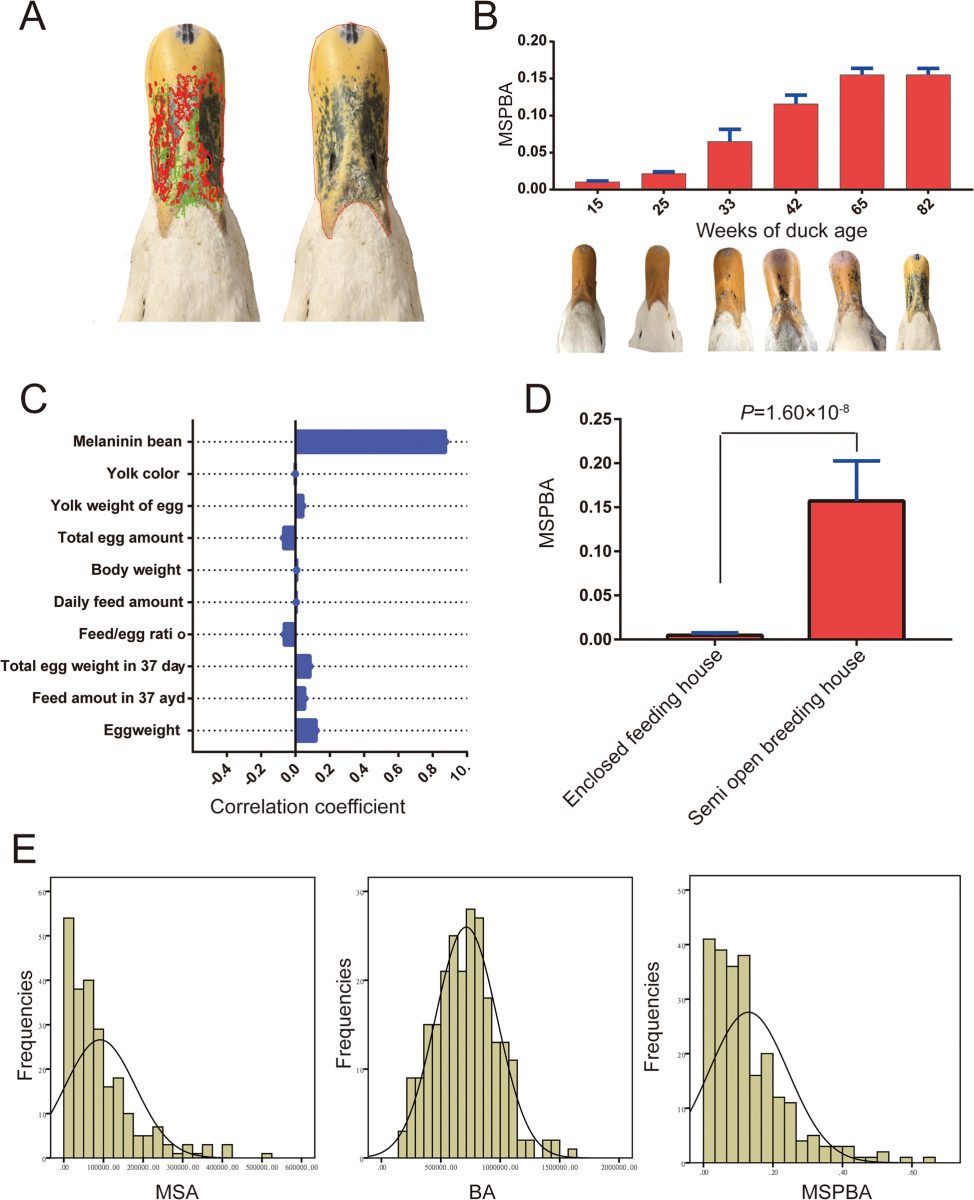
Phenotypic observations of melanin spot in duck beak skin. **A** IPP 6.0 was used for determining the melanin spot amount (MSA), beak area (BA), and the melanin spot amount per unit beak area (MSPBA). The red dot marked on the left beak showed the melanin spot distribution by the irregular function of IPP 6.0. The red line marked the beak on the right side in the region for calculating the beak area. **B** The melanin spot was accumulated accompanying the age of ducks. **C** The phenotypic correlations between MSPBA with reproduction-related traits. **D** The MSPBA was compared between ducks under an enclosed house (with no sunlight exposure during the growing period) and a semi-open breeding house (exposed to sunlight during the growing period). **E** The distribution states of phenotypic values including MSA, BA, and MSPBA from 223 ducks

We used the IPP 6.0 software to open the images and utilized its function to evaluate the amount of melanin spot and area of the duck beak (Fig. 1A). The melanin spot was mainly accumulated on the beak at the lateral growth period of ducks, and the amount of melanin spot increased gradually with age (Fig. 1B). However, the melanin spot on the duck's beak exhibited an individual difference. The correlations between melanin deposition in beak skin and egg production, egg weight, feed intake, and duck feed-egg ratio were analyzed to test the impacts of the reproduction process on melanin spot accumulation. The results showed that all correlations were less than 0.2 (Fig. 1C), indicating only slight relations between them. Whereas the melanin spot of ducks feeding under an enclosed house and a semi-open breeding house was compared, it was observed that the former ones had fewer melanin spots than the latter ones (Fig. 1D), suggesting the sunlight exposure may be the precise cause of melanin spot in duck beak skin. The phenotypic data of melanin spot in beak skin, beak area, and melanin deposition per unit beak area of 223 female individuals were collected. These phenotypes' values were approximately normal distribution (Fig. 1E, Table S1).

### Genome-wide association analysis (GWAS)

**Fig. 2:**
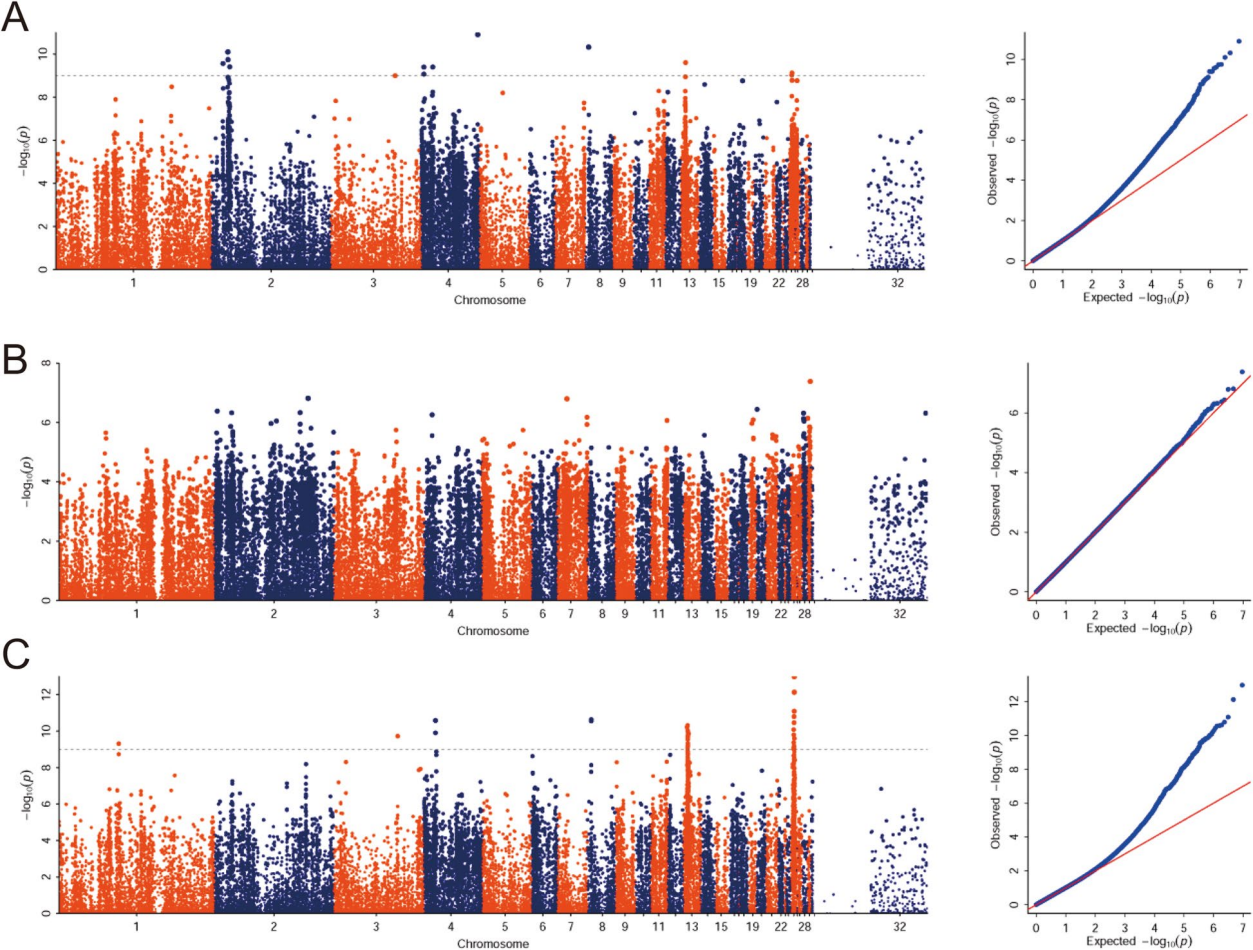
Manhattan (left) and QQ plots (right) show the significance of genetic effects on the Melanin spot in duck beak skin by a GWAS. **A** For melanin spot amount (MSA); **B** For beak area (BA); and **C** For melanin spot amount per unit beak area (MSPBA). The GWAS was performed in 223 F_2_ ducks crossed by Pekin ducks and mallards. The gray line represents the Bonferroni corrected significance threshold (-log_10_ *P* = 8.16). The x-axis shows the physical positions for each marker along the chromosomes, and the y-axis shows the -log_10_ *P* values for the association tests

We performed GWAS based on the genotypes and phenotypes, and the Manhattan and Quantile–Quantile (QQ) plots are shown in Fig. 2. For MSA, a single association peak was observed on chromosome 2 (*P* = 2.76 × 10^–10^; Fig. 2A), 13 (*P* = 2.5 × 10^–10^; Fig. 2A), and 25 (*P* = 7.59 × 10^–10^; Fig. 2A), respectively. For MSPBA, a single association peak was observed on chromosome 13 (*P* = 4.88 × 10^–11^; Fig. 2C) and 25 (*P* = 1.04 × 10^–13^; Fig. 2C), respectively. However, for the trait of BA, non-significant SNPs were observed (Fig. 2B). The QQ plots for MSA and MSPBA revealed that SNPs deviated from the distribution under the null hypothesis, which indicated a strong association between SNPs and phenotypes (Fig. 2). Therefore, the GWAS signals on chromosomes 13 and 25 for MSA were overlapped with the signals associated with MSPBA. As the signals of the later ones were stronger than the former ones, we thus continually focused on the GWAS signals of MSPBA. 46 SNPs on chromosome 13 and 25 on chromosome 25 reached the *bonferroni* corrected significance threshold (-log_10_ *P* = 8.16, Table S2 and Table S3) for this trait. These significant SNPs contributed a candidate genomic region ranging from 4.89 to 5.92 Mb on chromosome 13 and a region ranging from 3.35 to 4.47 Mb on chromosome 25, respectively (Fig. 2). For the GWAS signal for the MSA trait, 12 significant SNPs that reached the Bonferroni corrected significance threshold (-log_10_ *P* = 8.16) contributed a candidate genomic region ranging from 15.2 to 24 Mb on chromosome 2.

### Fine mapping the causative genomic region

**Fig. 3:**
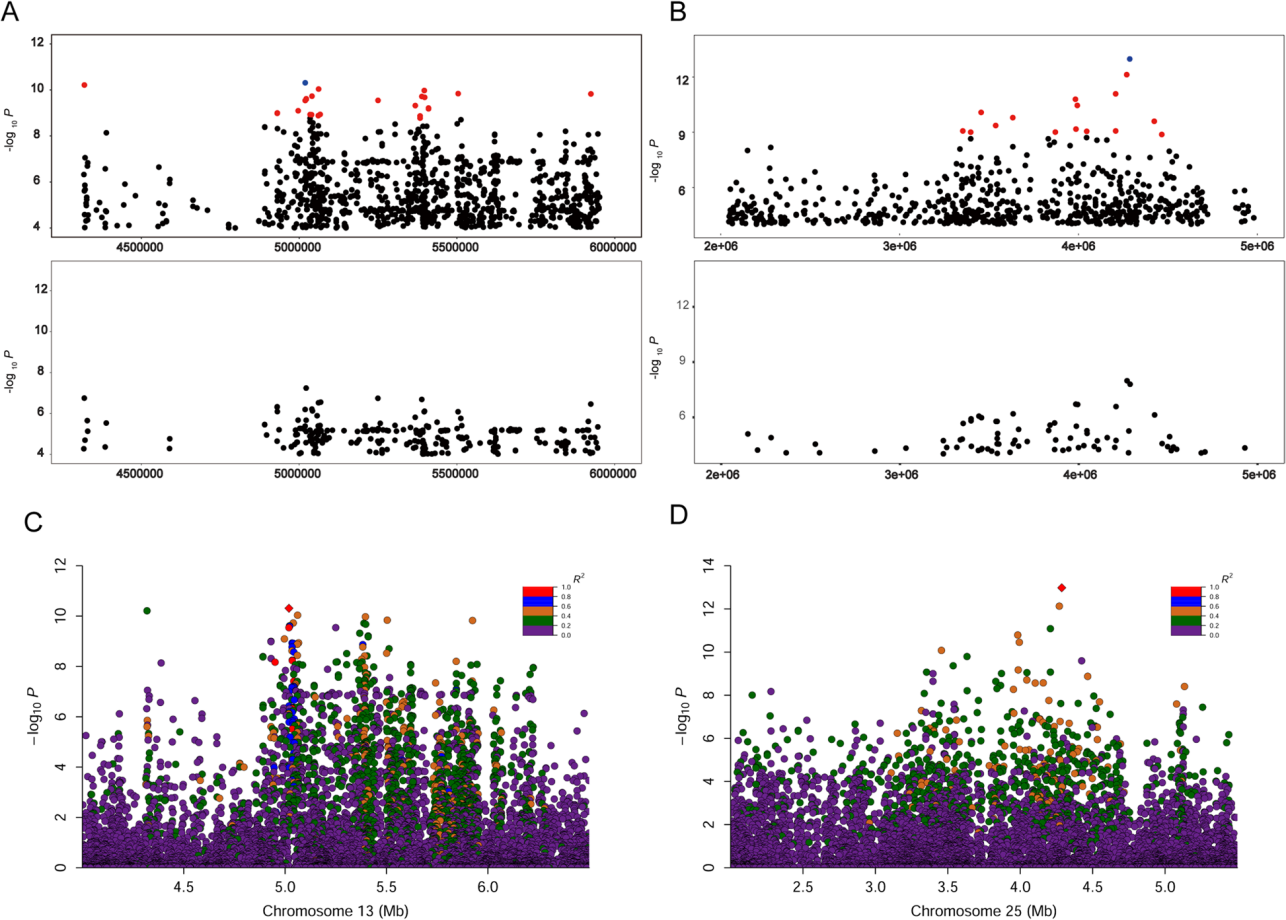
Conditional GWAS and regional plots of the loci associated with MSPBA. **A** and **B**. Conditional association analyses were carried out by fitting the most significant SNP Chr13:5,019,135 (*P* = 4.88 × 10^–11^) and Chr25:42,867,656 (*P* = 1.04 × 10^–13^) as a covariate for the melanin spot amount per unit beak area (MSPBA), respectively. **C** and **D**. Regional plots for the loci associated with MSPBA. All genotyped SNPs are color-coded according to their pairwise LD with the sentinel SNP (Chr13:5,019,135 and Chr25:42,867,656) calculated in the F_2_ intercross population. The strong LD block is defined as R^2^ ≥ 0.4

We conducted conditional GWAS of MSPBA using the loci in the candidate region by fitting the most significant SNP as a covariate factor (Fig. 3A and B). It was observed that the association peaks for these two signals have vanished. No more SNPs reached the significant threshold line of -log_10_ *P* = 8.16. The results implied high linkage relationships for these SNPs. For the signal on chromosome 13, the regional plots showed 27 significant SNPs have a higher pairwise LD that reached an R^2^ ≥ 0.4 with the leader significant SNP (Fig. 3C, Table S2). These SNPs in strongly pairwise LD supported a fine mapped region of 0.98 Mb ranging from 4.95 to 5.93 Mb on chromosome 13. For the signal on chromosome 25, the regional plots showed 11 significant SNPs have a higher pairwise LD that reached an R^2^ ≥ 0.4 with the leader significant SNP (Fig. 3D, Table S3). These significant SNPs were identified as being located in a ~ 1.0 Mb region spanning from 3.45 to 4.47 Mb on chromosome 25 because of the strong genetic correlations.

### Genes association with melanin spot deposition

**Fig. 4:**
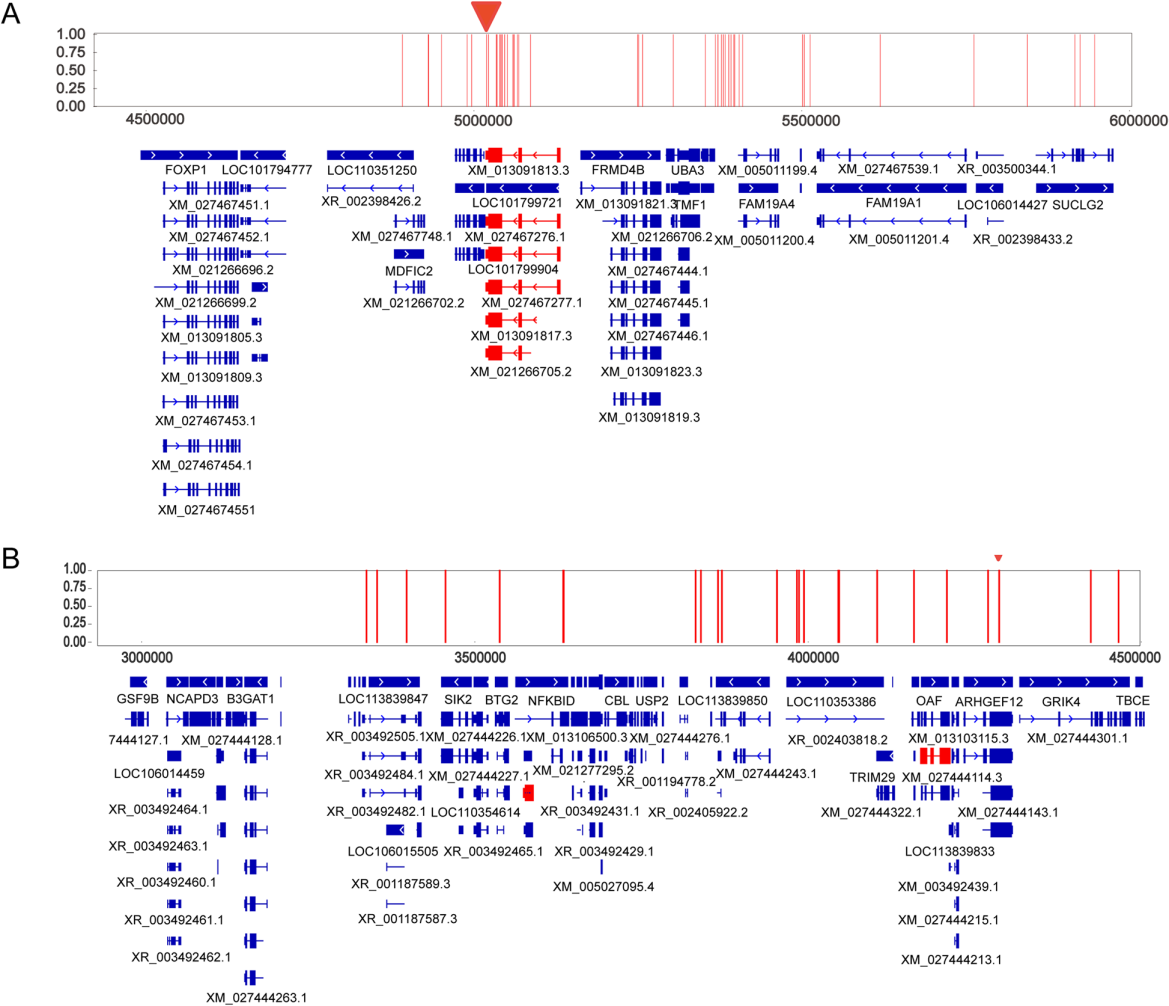
The distributions of significant SNPs and genes in the candidate chromosomal region. **A** The candidate genes in Chr13. **B** The candidate genes in Chr25. The reference duck genome was IASCAAS_Peking Duck_PBH1.5 (GCF_003850225.1), a top-level updated duck genome with N50 = 76,129,154, downloaded from NCBI. The triangle fulfilled in red represents the locations of the leader significant SNPs. Each vertical red line represents an SNP associated with melanin spot deposition that reached the significant threshold line of -log_10_ *P* = 8.16. The gene names in red were the potential candidate genes according to the expression profiles among tissues, supported by transcriptome from a global duck expression dataset

The fine-mapped region on chromosome 13 harbored 14 genes (Fig. 4A), which may play potential roles as candidate genes for the formation of melanin spots, including LOC101799721, LOC101799904, MITF, FRMD4B, LMOD3, ARL6IP5, UBA3, TMF1, EOGT, FAM19A4, LOC106014426, FAM19A1, LOC106014427, and SUCLG2. Among the 46 significant SNPs associated with MSPBA, 17 SNPs were distributed in the intron regions of the MITF gene. The leader significant SNP (Chr13: 5,019,135) in GWAS was located on the intron region of the MITF gene. Moreover, 7 SNPs located in the intron regions of the MITF have a higher pairwise LD that reached an R^2^ ≥ 0.7 with the leader significant SNP (Table S2). According to the global gene expression dataset based on the transcriptome, 10 out of 14 candidate genes were expressed in duck beak skin. Only MITF and LOC106014426 have a relatively higher expression level in beak skin than in other tissues (Fig. S1). The MITF was reported to play roles in melanocyte migration and melanin synthesis. Collectively, these data supported the MITF seemly as the causative gene for the formation of melanin spots in duck beak. The fine-mapped region on chromosome 25 harbored 42 candidate genes for the MSPBA trait. Three genes, including POU2F3, ARHGEF12, could be considered the candidate genes as they surrounded the leader significant SNP (Chr25: 4,286,765) in GWAS (Fig. 4B). According to the global gene expression dataset based on the transcriptome, 23 out of the 42 candidate genes expressed in duck beak skin with an average CPM value of more than 1. Among the 23 candidate genes, 17 were annotated as functional genes, including LAYN, POU2AF1, HINFP, NLRX1, RNF26, C1QTNF5, MFRP, USP2, THY1, NECTIN1, TRIM29, OAF, POU2F3, and ARHGEF12 (Fig. S2).

### A collaborate effects of MITF and POU transcription factors on melanin spot on duck beak skin

**Fig. 5:**
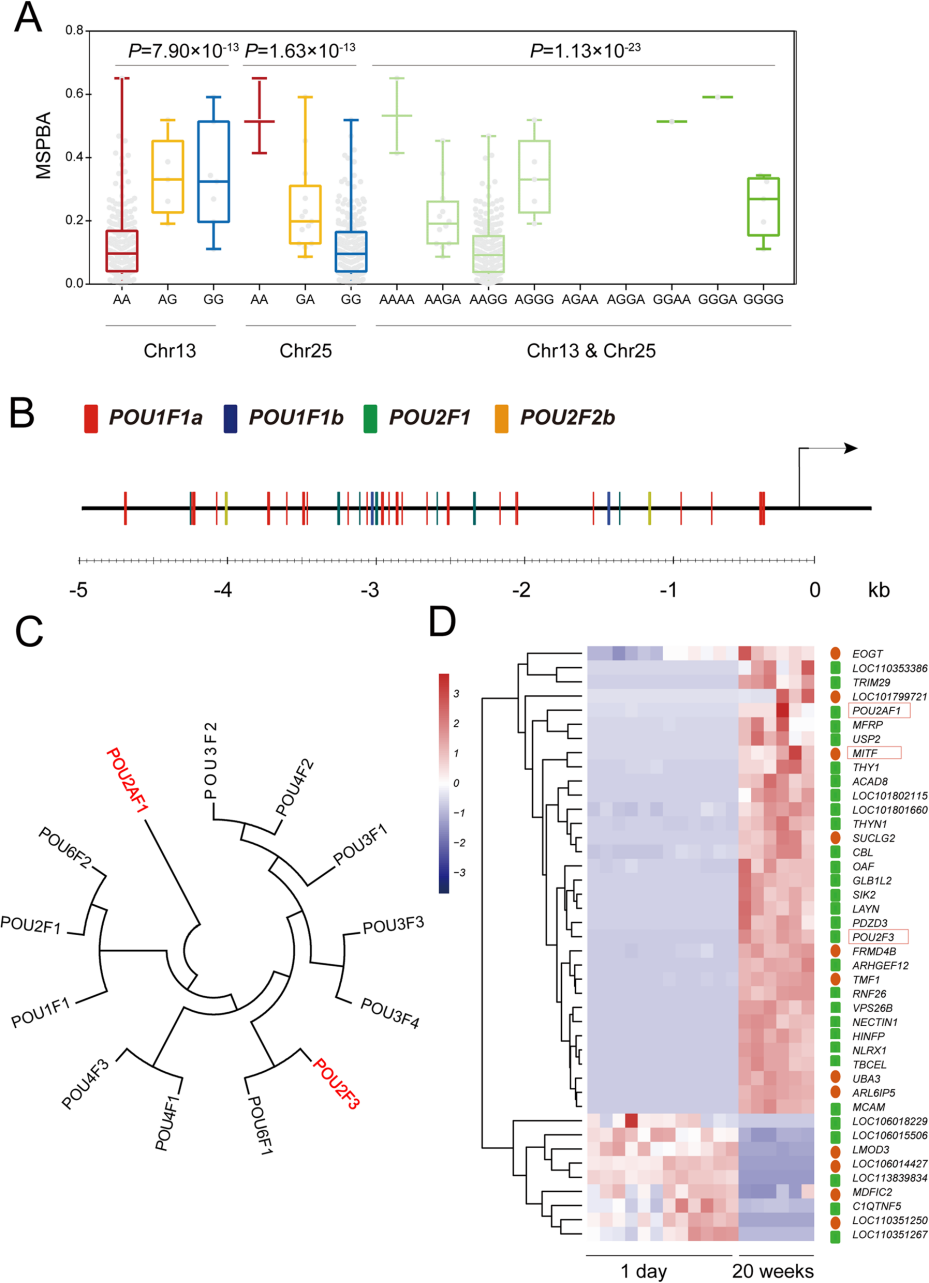
The potential regulatory relationships existed between MITF and POU transcription factors. **A** Box plot for effect of sentinel SNPs on Chr13 and Chr25 (Chr13:5,019,135 and Chr25:42,867,656) on MSPBA in 223 ducks. The indicated *P* values are based on one-way ANOVA. Box plots denote median (centerline), 25-75th percentile (limits), minimum and maximum values (whiskers). **B** the distribution of genomic locations of POU gene family transcription factors predicted within the cis-regulatory region, the sequence of 5 kb (Chr13:5,109,848–5,114,847) ahead of the transcription site of the MITF gene in duck genome. All POU transcription factors were provided in Table S4. **C** the molecular phylogenetic relationships of duck POU gene family members based on the Maximum Likelihood method. The gene members marked in red were predicted as the potential transcription factors of the duck MITF gene. **D** the cluster analysis of gene expression profiles of candidate genes. The circles fulfilled in red ahead of the gene names represent the genes located in Chr13, while the square fulfilled in green represents the genes located in Chr25

We genotyped all 223 ducks using allele information of the two leaders SNPs Chr13:5,019,135 and Chr25:42,867,656 and analyzed the impacts of genotypes by these two alleles on the phenotypes. These two leader SNPs can explain 17.6% and 22.3% for the phenotypic variance of MSPBA. The results demonstrated that these two loci had coordination effects on the MSPBA (Fig. 5A). These findings reminded us there might be regulatory relationships between the causative genes underlying the two GWAS signals, as some of the POU members were reported to play roles as transcription factors of the MITF gene. Therefore, we inferred there might be potential cis-regulatory relationships between the POU transcription factors underlying signal on chromosome 25 and the MITF gene underlying signal on chromosome 13. We thus downloaded the promoter sequence of the duck MITF gene, and the transcription factors and their binding sites were predicted by online software PROMO. As a result, 44 POU transcription factors were predicted to be distributed within the promoter region of duck MITF. These POU transcription factors were included POU1F1A, POU1F1B, POU2F1, and POU2F2B (Fig. 5B, Table S4). Although the candidate genes POU2AF1 and POU2F3, underlying the GWAS signal on chromosome 25, were not included in the four predicted POU transcription factors, they shared higher sequence homology with other POU members, according to a molecular phylogenetic tree by using all duck POU gene family members (Fig. 5C). Subsequently, a gene expression cluster analysis of the candidate genes on chromosomes 13 and 25 were performed using transcriptome data in the beak skin of ducks at the ages of one day old and twenty weeks old. The cluster results demonstrated that POU2AF1, POU2F3, and MITF have a similar expression pattern. All of them increased at the stage of 20 weeks old (Fig. 5D), suggesting combined effects on melanin spot deposition on duck beak skin.

## Discussion

Melanin is produced from tyrosine catalyzed by tyrosinase undergoing catalytic reactions [18]. Age and sunlight exposure are the main causes of the deposition of melanin spots on the human face and neck. This study found that melanin deposition in duck beak skin increased with age. Meanwhile, the melanin deposition in the beak skin of ducks reared under the fully enclosed house was lighter than the ducks feed in the semi-open house system. Our results were consistent with the study by Maddodi et al. [19] in humans, supporting that the melanin spot in the skin is related to age and sunlight exposure for both birds and mammals.

In poultry production, it was considered that the deposition of melanin in duck beak skin might be linked to reproductive activities. Previous studies have demonstrated an estrogen receptor in normal human melanocytes [20, 21]. Kim et al. [22] found that estrogen can promote the transport of melanocytes to keratinocytes, resulting in the formation of chloasma. Moreover, estradiol can induce deeper skin pigmentation at a definite concentration [23]. During the laying period of ducks, the melanin was deposited on the beak surface, and there were individual differences in the amount of melanin deposition. However, a low correlation was observed between melanin deposition in beak skin and reproduction abilities. Therefore, it suggested that the physiological, metabolic processes did not cause the melanin deposition in duck beak skin during reproductive activities.

In the GWAS analysis, to eliminate the effect of stratification, we have already added the fixed effects (PCA and the forward/backward cross effect) and random animal effects (kinship matrix estimated using the whole-genome SNP genotypes). The Q-Q results also confirmed a good GWAS result, as the scatter points in the first half show the rationality of the statistical model used, and the scatter points distributed above the diagonal in the second half of QQ show that loci related to traits have been found. Our final fine-mapping results identified that the MITF gene harbors in the GWAS signal on chr13, which is a crucial gene responsible for melanin synthesis. Therefore, the GWAS signal identified in this study is reliable.

Based on GWAS analysis, three loci associated with melanin amount were identified in the duck genome, and these loci were located on chromosomes 2, 13, and 25, respectively. Our findings implied that age, sunlight exposure, and genetic factors affected melanin deposition in duck beak skin. Similar results have also been reported in humans [12, 24]. We believed MITF and POU genes might be essential for melanin deposition in duck skin through further fine-mapping works and expression analysis. The MITF protein is considered a necessary regulator of melanocyte migration and differentiation [25]. Our findings implied that the melanin synthesis and metabolic pathway played a crucial role in inducing melanin deposition in the skin during age and sunlight exposure. Mutations in any genes related to melanin synthesis and metabolic pathway would affect melanin syntheses, such as MC1R, ENDRB, RAB27A, OA1, PMEL17, MLANA, GPNMB, Melastatin1, Aim1, TYR, TRP-1, TRP-2, and MART-1 [26]. Our findings in ducks also served as a reference for human beings to find the causative genes that affect melanin spots under sunlight exposure.

The POU was a transcription factor family member which can promote transcriptions of many genes related to development and metabolism [27]. This gene family set shares the typical POU domain structure [28]. POU derives from the major transcription factors described in the family [27]. POU members have been declared to regulate the transcription of the MITF gene [28, 29]. We found 13 POU gene family members within the duck genome. A total of 44 POU transcription factors were predicted to be distributed within the promoter region of the MITF, and they were POU1F1A, POU1F1B, POU2F1, and POU2F2B. In the candidate region of duck chromosome 25, we found two members of the POU gene family, namely POU2AF1 and POU2F3. The regulatory relationships between POU members and the MITF gene were also supported by the correlation analysis between allele frequencies and phenotypes, which suggested a coordination effect of these two loci on melanin deposition in duck beak skin. Collectively, our data indicated a collaboration effect of MITF and its POU transcription factors on melanin spot deposition on duck beak skin. However, their specific regulatory process needs to be further verified by more molecular biological evidence.

The variations in skin pigmentation result from adaptability to various geographical locations [24, 30]. The pivotal function of pigmentation is protecting the exposed skin against UV radiation [31]. Predictably, genetic variations within the genes involved in the pigmentation process caused phenotypic variations, such as skin pigmentation, hair color, eye color, freckling, and skin sensitivity to sunlight [32]. The ancestor of domestic ducks is the mallard, a kind of migratory bird, and the pigmentation variations in the skin are beneficial for adapting to a wide range of environmental conditions. We thus assumed that, after many mutations generated in the MITF and POU during the evolutionary process, the favorable mutations tended to be preserved in duck genomes to increase the adaptability to different light environments. However, accompanying with age and constant sunlight exposure, the asymmetrical distribution of melanocytes caused the formation of melanin spot in duck beak skin. In poultry production, some farmers believed that the melanin spot accumulation in beak skin of ducks is caused by fading of xanthophylls that cover the existing melanin. Nevertheless, our present study has not identified any genes, whose polymorphism related to xanthophylls synthesis were associated with the content of melanin in duck beak skins. The present study, laid new clues for understanding about genetic factors that can affect the melanin spot in the skin, however further investigation is required to strengthen this hypothesis.

## Conclusion

We demonstrated that age and sunlight exposure induces melanin deposition in duck beak skin, while heredity is fundamental. The MITF and POU2F3 likely played a synergistic effect on the regulation of melanin synthesis, and their mutations contribute to phenotypic differences in beak melanin deposition among individuals. It is pointed out that melanin deposition in the beak skin is related to the melanin metabolism pathway, which provides insights into the molecular regulatory mechanisms and the genetic improvement of the melanin deposition in duck beak.

## Materials and methods

### Animals

Total 223 F_2_ female ducks were used in this study from reciprocal crosses of Mallards and Pekin ducks. The Mallards were obtained from the Aoji Duck Farm (hunting license available, Zhejiang, China). Pekin ducks were kept on Pekin Duck Breeding Center of the Chinese Academy of Agricultural Sciences (Pekin, China). 100 female Mallards and 10 male Pekin ducks were selected for the orthogonal cross as parents. In the reciprocal cross, 4 male Mallards and 40 female Pekin ducks were selected as parents. For both cross-manner designs, one male duck was mated to 10 females. Thus, a total of 216 families was established using the F_1_ hybrids. The cross works were constructed in 2015, more than 2000 F_2_ ducks were obtained for subsequent studies. Most of them were slaughtered for growth performance studies [17]. The left ducks were sequentially raised under the same conditions in individual cages for phenotype measurement in this current study.

### Genotyping

Blood samples were collected from the wing vein of female ducks. 223 blood samples were selected for DNA extraction using the phenol–chloroform protocol. The quality and quantity of DNA were examined by Nanodrop and agarose gel electrophoresis. The paired-end libraries were generated for each eligible sample using standard procedures. Besides, the average insert size was 500 bp, and the read length was 150 bp. All libraries were sequenced on an Illumina®Hiseq X-Ten platform in a Bio-company (Berry Genomics, Pekin, China) to average raw read sequence coverage of 5 × for a paired-end of 150 bp (PE150).

The raw reads were filtered using NGS QC (v2.3.3) Toolkit with default parameters [33]. The clean reads were mapped to the duck reference genome (IASCAAS_PekingDuck_PBH1.5, GCF_003850225.1) with Burrows-Wheeler alignment (BWA aln) [34] using the default parameters. Next, SNPs calling was performed using GATK (version 3.5) exclusively [35], and the output was further filtered using VCFtools (version 0.1.15) [36]. The SNPs were screened with the parameters of a minor allele frequency (MAF) > 0.05, a max allele frequency < 0.99, and the maximum missing rate was < 0.1. After filtering, 9,584,532 SNPs remained with a mean density of 8.5 SNPs/kb across the genome. All filtered SNPs were distributed on 29 autosomal chromosomes, Chr Z, Chr W, and Chr U (unplaced scaffolds).

### Phenotypes` collection

Melanin spots in the beak skin of all ducks were photographed in the same conditions. First, the digital camera was set to a manual exposure manner, and each photo was based on identical exposure conditions, including exposure time and aperture. Then, the obtained images were imported into IPP 6.0 software (Media Cybernetics, USA), and afterward, they were magnified by the identical multiple. Next, using an irregular tool incorporated in the software, the area of interest (AOI) of each melanin spot was selected, and the geometric size of each region was measured. Finally, melanin spot area in duck beak skin (MSA) and beak area (BA) was measured, respectively, and the melanin distribution per unit area of beak skin (MSPBA) was calculated. Three replicates in each measurement and the average values were taken as the final phenotype.

To compare the melanin spots of duck beak skin among ages, 10 Pekin ducks were randomly selected at each age time point. Total 30 ducks at 60-week-old were randomly chosen from fully enclosed house feeding (mainly with artificial light only instead of whole sunlight exposure during the growing period) and a semi-open breeding house (exposed to sunlight during the growing period), respectively, and melanin spots in beak skin of them were determined by the method mentioned above. All other feeding management conditions were kept the same. The primary purpose was to compare the effect of sunlight on the deposition of melanin spots in beak skin. In addition, the reproduction abilities of 223 F_2_ female ducks were determined by egg production and feed conversion ratio.

### Whole-genome association analysis

GWAS was performed on the F_2_ population to detect genomic regions that affect melanin spot traits in ducks, including melanin spot amount (MSA), beak area (BA), and melanin spot amount per unit beak area (MSPBA). The GWAS was analyzed using the mixed linear model program EMMAX [37]. The linear model used to test each SNP individually was:

1 $$\mathrm{y}=\mathrm{X\alpha }+\mathrm{Z\beta }+\mathrm{W\mu }+\mathrm{e},$$

Where $\mathrm{y}$ is the vector of observed phenotypes, including melanin spot amount (MSA), beak area (BA), and melanin spot amount per unit beak area (MSPBA); $\mathrm{X\alpha }$ represents the fixed effects, including the first three principal component values (PCA eigenvectors) derived from the whole-genome SNP genotypes, to correct population stratification, and the forward/backward cross effect; $\mathrm{Z\beta }$ represents the effect of the tested SNP, with allele substitution effect $\upbeta$; $\mathrm{W\mu }$ represents the random animal effect, with a variance–covariance structure based on the kinship matrix estimated using the whole-genome SNP genotypes.$\mathrm{e}$ is the vector of random residual errors. SNPs with a *p*-value that reached a *Bonferroni*-corrected threshold (− Log_10_ (*P*) ≥ 8.16) were considered significant.

### Gene expression analyses

Duck global expression dataset of mallards and Pekin ducks has been constructed previously [17], where a global transcriptome project of ducks was conducted. Total 10 tissues were include in the database, including beak skin, lung, abdominal fat, liver, brain, skin, spleen, kidney, heart and muscle. First, the clean reads of RNA-seq paired-end reads were mapped against the Pekin duck reference genome (IASCAAS_PekingDuck_PBH1.5, GCF_003850225.1) using the Tophat read mapper. Then Htseq-count were run for reads count per gene and calculated the CPM (counts per million) value of genes.

Genes with similar functions or regulatory relations usually exhibit identical expression patterns. To demonstrate the potential regulatory effects of the predicted transcription factors on MITF transcriptions, their expression levels in beak skin based on transcriptome data of 18 individuals were collected for gene expression clusters [38]. These 18-transcriptome data were performed in the beak skin of the ducks at ages of 1 day old (*n* = 12) and 20 weeks old (*n* = 6).

### Transcription factors’ prediction

The online software PROMO (http://alggen.lsi.upc.es/cgi-bin/promo_v3/promo/promoinit.cgi?dirDB=TF_8.3) was used for predicting transcription factors and their binding sites on the cis-regulatory region of the duck MITF gene. The 0.5 kb (Chr13:5,109,848–5,114,847) promoter sequence of duck MITF gene was intercepted for bioinformatical analysis.

### Molecular evolution analysis

The coding region sequence (CDS) of each duck POU gene family member was downloaded from NCBI. There was a total of 13 lines in the duck genome (IASCAAS_PekingDuck_PBH1.5, GCF_003850225.1). Evolutionary analyses were conducted in MEGA7 [39]. The evolutionary relations were inferred using the Maximum Likelihood method based on the Hasegawa-Kishino-Yano model [40]. The bootstrap consensus tree inferred from 1000 replicates [41] was taken to represent the evolutionary history of the taxa analyzed [41]. Branches corresponding to partitions reproduced in less than 50% bootstrap replicates are collapsed.

### Data statistic

One-way ANOVA analyzed all the data with the SPSS19.0 software package. Differences at *p* < 0.05 were considered significant.

## Supplementary Information

**Additional file 1. **

**Additional file 2. **

**Additional file 3. **

**Additional file 4. **

**Additional file 5. **

**Additional file 6. **

## Data Availability

The Genome sequencing raw data was available in NCBI's Sequence Read Archive database (Accession number: CRA003272). The RNA sequencing raw data was previously released in NCBI's SRA database (Accession number: PRJNA471401 and PRJNA450892).

## Abbreviations

GWAS: Genome-wide association studies
UV: Ultraviolet rays
MITF: Melanocyte Inducing Transcription Factor
POU2F3: POU class 2 homeobox 3
MC1R: Melanocortin 1 Receptor
α-MSH: Alpha-melanocyte-stimulating hormone
MAF: Minor allele frequency
AOI: The area of interest
MSA: Melanin spot area in duck beak skin
BA: Beak area
MSPBA: The melanin distribution per unit area of beak skin
CDS: Coding region sequence
LD: Linkage disequilibrium
SNP: Single-nucleotide polymorphism
