# Supplementary figures and images for: Genome-wide association analysis reveal the genetic reasons affect melanin spot accumulation in beak skin of ducks
Source: BMC Genomics. 2022 Mar 26;23:236. doi: 10.1186/s12864-022-08444-5 (PMC8962612; doi:10.1186/s12864-022-08444-5)

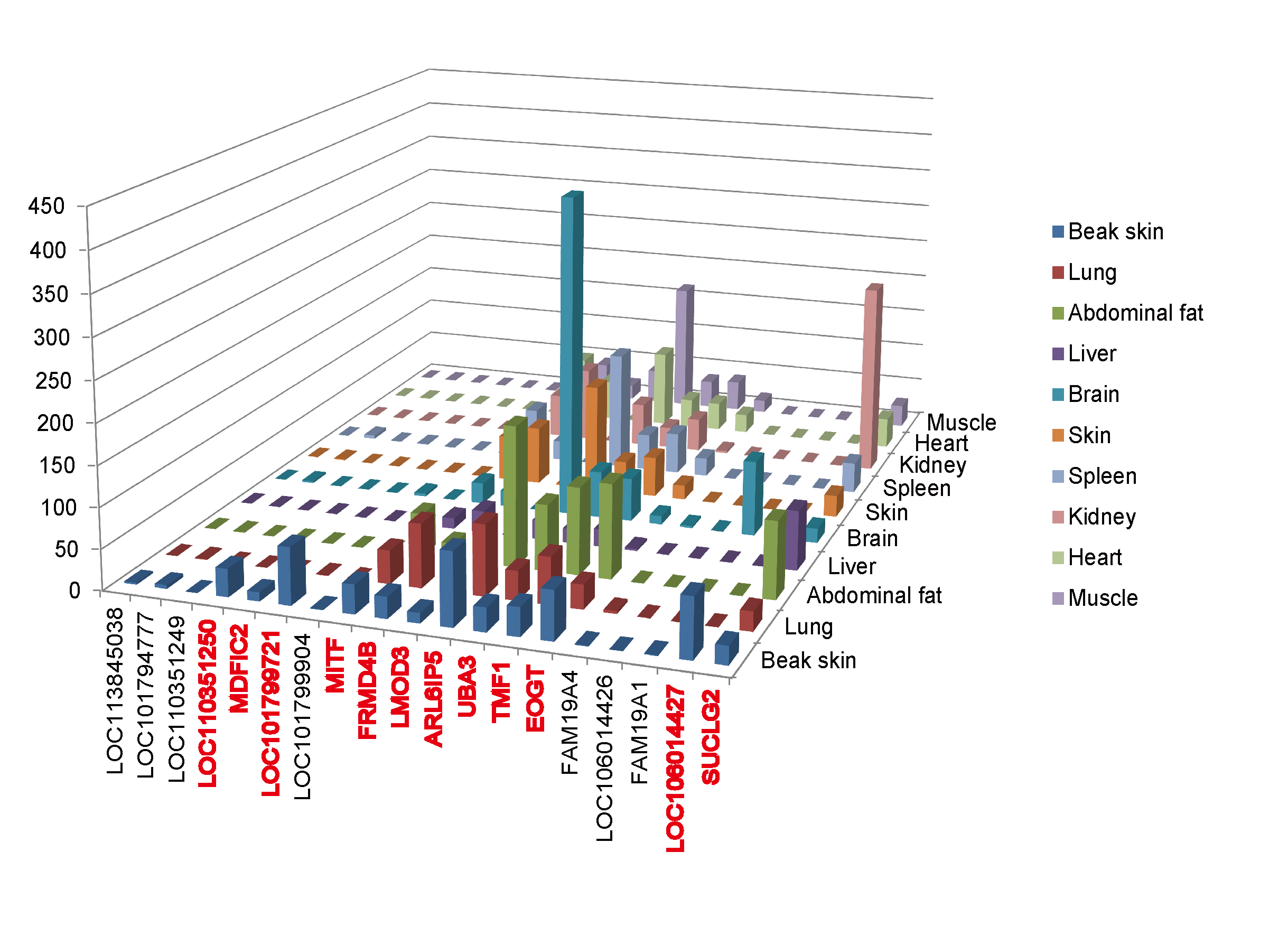

Supplement: Supplementary file 1 — Additional file 1. [file 12864_2022_8444_MOESM1_ESM.tif]

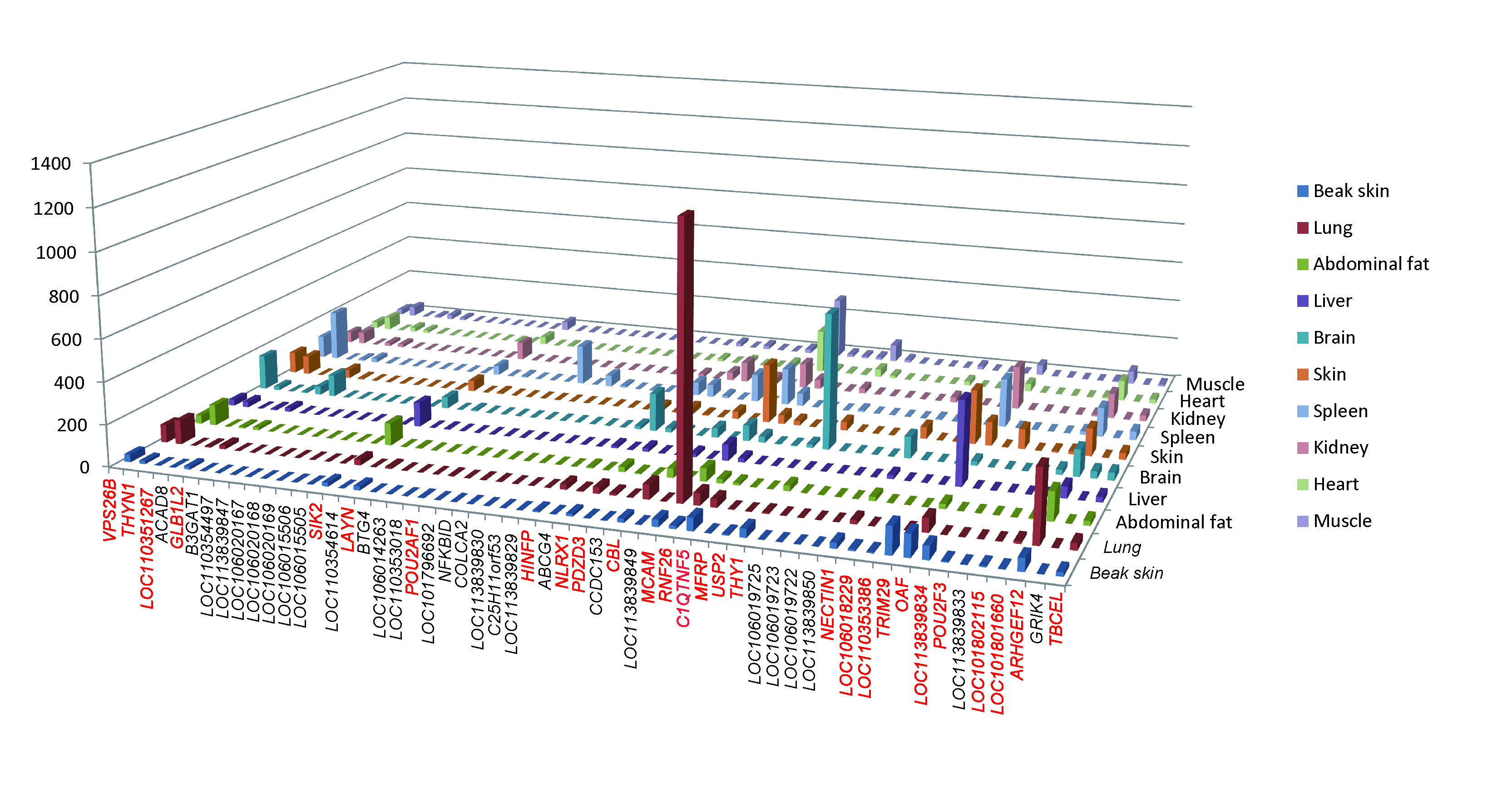

Supplement: Supplementary file 2 — Additional file 2. [file 12864_2022_8444_MOESM2_ESM.tif]
