# Supplementary material for: Genome-wide association analysis reveal the genetic reasons affect melanin spot accumulation in beak skin of ducks
Source: BMC Genomics. 2022 Mar 26;23:236. doi: 10.1186/s12864-022-08444-5 (PMC8962612; doi:10.1186/s12864-022-08444-5)
Supplement: Supplementary file 3 — Additional file 3. [file 12864_2022_8444_MOESM3_ESM.docx]

**Table S4.** Descriptive statistics for MSA, BA and MSPBA traits in F_2_ offspring populations

| **Traits** | **N** | **Mean** | **S.D.** | **Min** | **Max** | **Median** | **Skewness** | **Kurtosis** |
| --- | --- | --- | --- | --- | --- | --- | --- | --- |
| MSA | 223 | 84370.28 | 78528.73 | 344.00 | 416236.67 | 63376.00 | 1.66 | 3.12 |
| BA | 223 | 717287.78 | 251528.79 | 165125.33 | 1624265.66 | 716975.33 | 0.31 | 0.29 |
| MSPBA | 223 | 0.12 | 0.10 | 4.17 | 0.65 | 0.09 | 1.70 | 4.29 |
